# Progressive Stenosis of Thoraflex Hybrid Prosthesis after Total Arch Replacement Leading to a Fatal Outcome: A Case Report

**DOI:** 10.3400/avd.cr.25-00094

**Published:** 2025-12-09

**Authors:** Norimasa Haijima, Mikihiko Kudo, Satoru Murata, Takuya Ono, Hideyuki Shimizu

**Affiliations:** 1 Department of Cardiovascular Surgery, National Hospital Organization Saitama Hospital, Wako, Saitama, Japan; 2 Department of Cardiovascular Surgery, Keio University Hospital, Tokyo, Japan

**Keywords:** Thoraflex Hybrid, graft stenosis, total arch replacement

## Abstract

The Thoraflex Hybrid prosthesis (Terumo Aortic, Inchinnan, UK) is widely used for total arch replacement, but postoperative stenosis or pseudo-coarctation is rare and potentially fatal. We report an 83-year-old man who underwent a Bentall procedure and total arch replacement with a 36-mm Thoraflex Hybrid graft (Terumo Aortic). Postoperative imaging showed mild stenosis, but distal perfusion was maintained. Eighteen hours later, his cardiac index dropped to 1.4 L/min/m^2^ and lactate rose to 11.2 mmol/L. Computed tomography (CT) revealed severe graft stenosis. Although rescue thoracic endovascular aortic repair (TEVAR) transiently improved hemodynamics, the patient succumbed to multiorgan failure despite veno-arterial extracorporeal membrane oxygenation (VA ECMO). This case underscores the need for early recognition and prompt intervention.

## Introduction

The frozen elephant trunk (FET) technique has become a standard treatment for complex aortic arch aneurysms and dissections. Recent advancements in branched and hybrid prostheses have significantly improved surgical outcomes. The Thoraflex Hybrid (Terumo Aortic, Inchinnan, UK), which combines a branched graft and a stent graft, is now widely used for total arch replacement. While rare cases of postoperative complications such as graft kinking or incomplete stent expansion leading to pseudo-coarctation have been reported, these were typically detected intraoperatively and successfully managed.

We report a rare case of progressive stenosis following a Bentall procedure and total arch replacement using the Thoraflex Hybrid prosthesis (Terumo Aortic). Unlike previous reports, the stenosis developed postoperatively and was associated with a torsion-prone descending aorta, use of the pull-through delivery technique, and the relatively low radial force of the device. Despite initial preservation of distal perfusion, the progressive narrowing culminated in multiorgan failure and death. This case underscores that kinking can evolve even after an apparently successful implantation, highlighting the importance of early recognition and preparedness for urgent endovascular rescue.

## Case Report

An 83-year-old man with a medical history of hypertension and dyslipidemia was referred to our hospital after an abnormal chest shadow was detected during a routine health check. Laboratory tests revealed an elevated N-terminal pro-B-type natriuretic peptide (NT-proBNP) of 2297 pg/mL, with other values being within normal limits. Transthoracic echocardiography showed a left ventricular end-diastolic diameter of 58 mm, an end-systolic diameter of 42 mm, and an ejection fraction of 49.8%, indicating mildly reduced systolic function. He had severe aortic regurgitation (AR), as well as mild mitral and tricuspid regurgitation (MR and TR). The regurgitant jet was centrally directed, and the left ventricular end-diastolic volume index was 93.6 mL/m^2^. Regional wall motion at the apex was reduced.

**Fig. 1:**
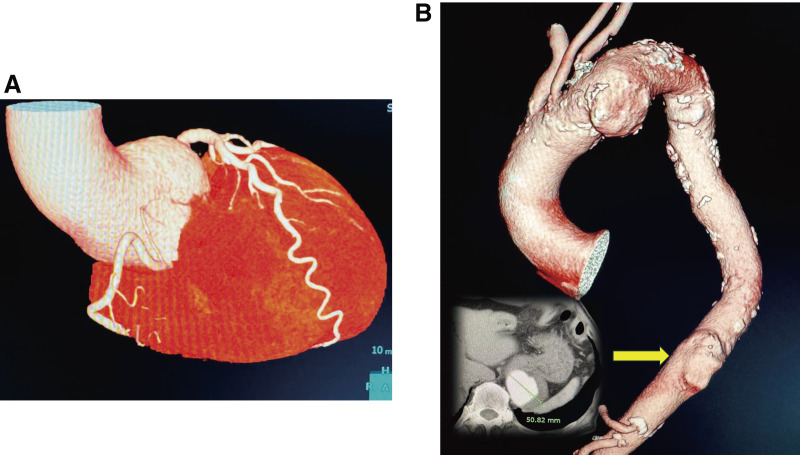
Preoperative imaging. (**A**) A contrast-enhanced CT scan showing dilation of the sinus of Valsalva and ascending aorta, with a maximum diameter of 60 mm. (**B**) A contrast-enhanced CT scan demonstrating a saccular aneurysm in the lower part of the aortic arch (arrow), measuring approximately 50 mm, with mild calcification and mural thrombus. CT: computed tomography

Coronary angiography showed no significant stenosis. Contrast-enhanced computed tomography (CT) revealed dilatation extending from the sinus of Valsalva to the ascending aorta, with a maximum root diameter of 60 mm and loss of the sinotubular junction (**Fig. 1A**). A saccular aneurysm measuring approximately 50 mm was also identified in the inferior portion of the aortic arch (**Fig. 1B**). This aneurysm had mild calcification and a 2–3-mm-thick mural thrombus. Additionally, a 40-mm saccular aneurysm was present in the descending thoracic aorta. On centerline-orthogonal cut-planes, the lumen long-axis orientation rotated progressively from the distal arch toward the proximal descending aorta (θ: 134°, 141°, 21°, 53°; cumulative Δθ ≈ 99°), with ellipticity (b/a 0.72–0.84), indicating a torsion-prone segment distal to the left subclavian artery (LSA).

Based on these imaging findings, the patient was diagnosed with annuloaortic ectasia accompanied by multiple thoracic aortic aneurysms. Surgical treatment was indicated. The patient underwent a Bentall procedure with a bioprosthetic valve and a total arch replacement using a 36-mm Thoraflex Hybrid prosthesis (Terumo Aortic).

### Intraoperative findings

Prior to arch replacement, the left axillary artery was exposed for left subclavian artery reconstruction. After systemic heparinization (activated clotting time >250 seconds), a 7-mm straight J-graft (Japan Lifeline, Tokyo, Japan) was anastomosed to the artery in an end-to-side fashion.

A 36 × 150-mm Thoraflex Hybrid prosthesis (Terumo Aortic) was selected for the total arch replacement. To ensure accurate deployment, the right femoral artery was punctured with a Micropuncture Introducer Set (Cook Medical, Bloomington, IN, USA), and a 0.035-inch Radifocus Guidewire M (Terumo, Tokyo, Japan) was advanced into the ascending aorta.

Following median sternotomy, the pre-anastomosed graft was introduced into the pericardium. Cardiopulmonary bypass (CPB) was established via ascending aortic arterial cannulation and single right atrial venous cannulation. A composite graft was then constructed using a 28-mm Valsalva graft (Terumo Aortic) and a 23-mm Inspiris Resilia bioprosthetic valve (Edwards Lifesciences, Irvine, CA, USA). After aortic cross-clamping and cardioplegic arrest, the ascending aorta was incised, the guidewire was secured, and the Bentall procedure was performed. Systemic cooling was initiated to a target nasopharyngeal temperature of 27°C.

For selective cerebral perfusion, the left axillary artery graft was connected to the cerebral perfusion circuit, and the left subclavian artery was ligated at its origin. Under circulatory arrest, the innominate and left common carotid arteries were clamped, and the aorta was incised at Zone 1. The Thoraflex Hybrid was inserted using a pull-through technique, and the distal anastomosis was completed. Lower body perfusion was then resumed through the side branch, and proximal anastomosis with the Valsalva graft was performed. Rewarming was initiated, followed by sequential reconstruction of the supra-aortic branches.

The patient was successfully weaned from CPB. Transesophageal echocardiography revealed incomplete expansion of a portion of the stent graft, but no significant pressure gradient between the upper and lower extremities was observed. Systemic blood pressure and blood gas values remained stable, and the procedure was completed without further complications.

The total operative time was 361 minutes, CPB time was 205 minutes, and circulatory arrest time was 30 minutes.

### Postoperative course

#### Immediate postoperative period (0–6 hours)

**Fig. 2:**
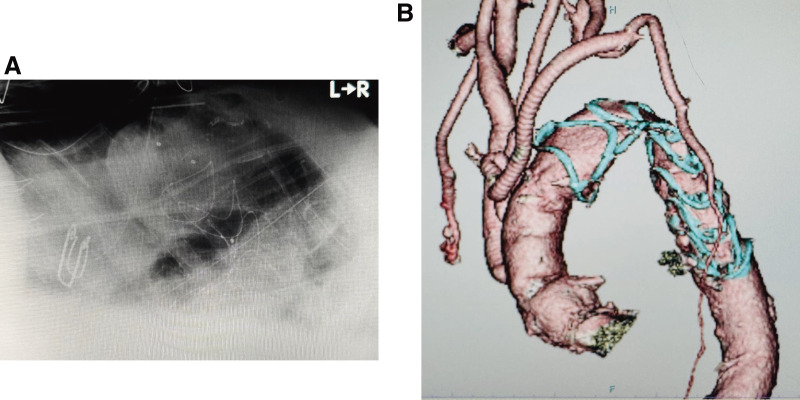
Early postoperative imaging. (**A**) A postoperative chest radiograph indicating potential kinking and stenosis of the arch stent graft. (**B**) A contrast-enhanced CT scan from POD 1, confirming severe stenosis of the Thoraflex Hybrid prosthesis (Terumo Aortic, Inchinnan, UK) in the aortic arch. CT: computed tomography; POD: postoperative day

Postoperative chest radiography suggested kinking and stenosis of the arch stent graft, although lateral views excluded complete obstruction (**Fig. 2A**). A systolic blood pressure difference of approximately 10 mmHg was noted between the upper and lower extremities, but peripheral perfusion appeared preserved with palpable pedal pulses. Upon admission to the intensive care unit, vital signs and arterial blood gas values were acceptable. However, the cardiac index (CI) was low at 1.6 L/min/m^2^, and mixed venous oxygen saturation decreased to 42% within 3 hours, indicating progressive systemic hypoperfusion. No postoperative bleeding was observed.

#### Postoperative day 1 (POD 1)—Rescue TEVAR

**Fig. 3:**
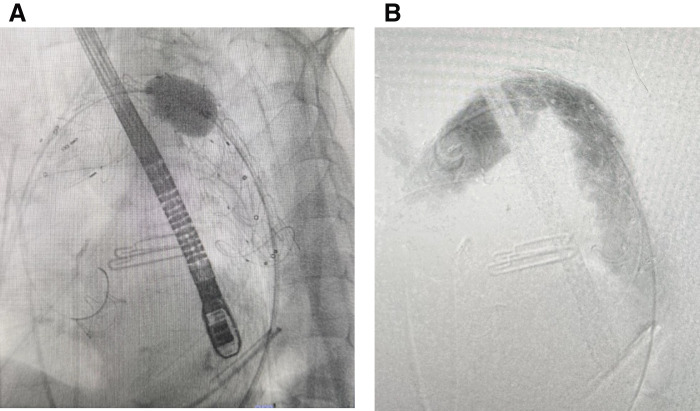
Rescue TEVAR findings. (**A**) An intraoperative angiogram taken before the rescue TEVAR procedure, which shows significant stenosis at the arch portion of the Thoraflex graft (Terumo Aortic, Inchinnan, UK). (**B**) An angiogram obtained after balloon dilation with a Reliant Balloon (Medtronic, Minneapolis, MN, USA) demonstrating the resolution of the stenosis and the subsequent disappearance of the blood pressure gradient between the upper and lower extremities. TEVAR: thoracic endovascular aortic repair

The CI further declined to 1.4 L/min/m^2^, with lactate levels rising from 8.3 to 11.2 mmol/L. The patient remained alert and responsive but developed motor weakness in the lower extremities. Contrast-enhanced CT revealed severe stenosis of the Thoraflex graft at the aortic arch, distal to the LSA, which had progressed from the immediate postoperative findings (**Fig. 2B**). Under a standardized left anterior oblique (LAO) 35° projection, representative bend angles at 3 predefined landmarks (proximal ascending, mid-ascending, pre-brachiocephalic artery [BCA]) were 158°/128°/149° preoperatively versus 152°/142°/152° postoperatively, indicating no material postoperative steepening of the proximal arch. To restore flow while minimizing spinal cord ischemia risk, targeted re-lining of the stenotic segment was performed with a stent-graft length similar to the distal FET segment, deliberately avoiding long-segment descending coverage. Therefore, rescue thoracic endovascular aortic repair (TEVAR) was performed using a Valiant Thoracic stent graft (VAMF3636C150TJ; Medtronic, Minneapolis, MN, USA) (**Fig. 3A**). Adjunctive balloon dilation with a Reliant Balloon (Medtronic) successfully relieved the stenosis, eliminating a pressure gradient of approximately 30 mmHg between the proximal and distal sites (**Fig. 3B**). Intraoperative transesophageal echocardiography showed a reduced ejection fraction of approximately 30%. Although intra-aortic balloon pumping (IABP) was considered, it was deferred at this stage, given the adjacent descending aneurysm and acceptable systemic pressure.

#### PODs 2–4—Development of organ dysfunction

Although lactate levels temporarily decreased after TEVAR, the CI remained low, ranging between 1.0 and 1.6 L/min/m^2^ and reaching a nadir of 1.0 on POD 7. On POD 4, liver enzymes rose sharply (aspartate transaminase 11230 U/L; alanine transaminase 2662 U/L), consistent with ischemic hepatitis (shock liver). Coagulopathy was also evident, with a PT-INR of 3.17 and an activated partial thromboplastin time of 102 seconds at POD 3 + 6 hours, suggesting disseminated intravascular coagulation (DIC). Continuous hemodiafiltration (CHDF) was continued from POD 2 to POD 11.

#### POD 3—Circulatory collapse (VA ECMO)

With recurrent hyperlactatemia and worsening circulatory failure, veno-arterial extracorporeal membrane oxygenation (VA ECMO) was initiated. A distal perfusion cannula was added to prevent limb ischemia, but blood pressure remained unstable. Echocardiography demonstrated a severely reduced ejection fraction of 13%. To augment support under ECMO, an IABP was subsequently introduced despite the presence of a descending thoracic aneurysm.

#### PODs 5–10—Progression to multiorgan failure

Although some parameters showed transient improvement, cholestasis progressed, with total bilirubin rising to 26.8 mg/dL on POD 9. Platelet counts dropped to 20000/μL, and inflammatory markers remained elevated (white blood cell [WBC] 23.8 × 10^3^/μL on POD 9). The patient remained CHDF-dependent, with a blood urea nitrogen level of 42.4 mg/dL on POD 10. Mixed venous oxygen saturation temporarily improved to 68.3% on POD 6, but organ dysfunction did not reverse, and lactate remained elevated at 6.02 mmol/L on POD 10.

#### POD 12—Outcome

Persistent circulatory failure and multiorgan dysfunction culminated in the patient’s death on POD 12. The clinical course and prognosis were explained to the family, and the patient passed away with their consent for palliative care.

## Discussion

The FET technique is a standard strategy for complex arch and proximal descending aortic disease, with contemporary series reporting favorable outcomes.^1–3)^ Nevertheless, rare device-related complications such as kinking or stenosis do occur.^4–6)^ Our case is notable for postoperative and progressive stenosis in the arch portion of a Thoraflex Hybrid graft, culminating in low-output syndrome and multiorgan failure despite transient hemodynamic improvement after rescue TEVAR.

### Mechanism

This event is interpreted as a multifactorial interaction among (i) a torsion-prone segment distal to the LSA—as quantified in the Case Report (cumulative Δθ ≈ 99°, b/a 0.72–0.84), (ii) the use of a stabilizing pull-through delivery to ensure distal landing (with the guidewire withdrawn before deployment), and (iii) the ring-stent radial-force profile of the hybrid device. Prior reports link FET kinking to deployment at vascular flexures, length/positioning issues, and intra-procedural twist.^4,7)^ Balloon dilation has been life-saving in cases of acute obstruction.^6)^ In a torsion-biased segment, reduced pre-deployment conformability from a body-floss approach may lower the threshold for rotational kinking when combined with comparatively lower radial force.^4,7)^

### Device design

The Thoraflex Hybrid’s ring-stent architecture provides comparatively lower radial force than Z-stent constructs—advantageous in acute dissection but potentially less resistant to extrinsic vectors in aneurysmal segments—thereby predisposing to compression, migration, and kinking.^4,7)^ Conversely, alternative designs may develop junctional kinking at the stented/non-stented interface when the non-stented segment is long; post-deployment ballooning is recommended in that setting.^4)^ In our patient, Frozenix (Japan Lifeline) was not feasible because of a diameter mismatch, so the complication is best regarded as multifactorial rather than a device-specific failure.

### Emergency TEVAR and IABP

Management was tailored to the patient’s anatomy and physiology. Short, targeted re-lining with a length similar to the distal FET segment was performed to relieve the focal obstruction while avoiding long-segment descending coverage and potential spinal cord ischemia; adjunctive balloon dilation abolished the ≈30 mmHg gradient (**Fig. 3**) and transiently improved perfusion. IABP was initially deferred given the adjacent descending aneurysm and concerns about device–device interaction, and was used later as an adjunct under VA-ECMO when circulatory collapse ensued.

### Impella feasibility

Contemporary guidance emphasizes individualized anatomical assessment and access-route selection for mechanical circulatory support (MCS) rather than numeric tortuosity cut-offs; indications and operation are addressed within broader percutaneous cardiopulmonary support (PCPS)/ECMO/Impella frameworks.^8)^ In our setting—tortuous arch/descending geometry, recent total arch replacement with limited axillary options, and a neighboring aneurysm—we judged large-bore arterial MCS high-risk and prioritized definitive relief of the mechanical obstruction with TEVAR.

### Bentall-related morphology and LSA bypass

Pre- and postoperative LAO 35° measurements showed no material postoperative steepening of the proximal arch, supporting that the driver resided distal to the LSA rather than within the ascending segment. A 1-piece multibranched arch prosthesis was used; the extra-anatomic LSA–axillary bypass did not alter the length or compliance of the proximal non-stented segment within the mediastinum. While a small contributory effect cannot be excluded, its role appears secondary to the distal torsion-biased geometry, delivery approach, and device mechanics.

### Clinical implication

Taken together with the immediate elimination of the pressure gradient after re-lining, these observations indicate that graft stenosis—not primary ventricular failure—dominated the hemodynamic picture. Even when intraoperative findings appear acceptable, delayed rotational kinking can still evolve. Vigilant early imaging, readiness for short, targeted endovascular re-lining, and judicious selection of circulatory support are critical to outcomes.^4–7,8)^

## Conclusion

This rare case highlights progressive, ultimately fatal post-FET stenosis after Thoraflex Hybrid implantation, despite transient improvement following rescue TEVAR. Postoperative low-output and hypoperfusion should prompt consideration of device-related stenosis rather than attribution to primary heart failure alone. Early recognition, prompt endovascular correction, and judicious circulatory support are critical.
